# Relationship between cortical diffusivity and neuropathological substrates in Alzheimer’s disease

**DOI:** 10.1002/alz.093309

**Published:** 2025-01-03

**Authors:** Justine Debatisse, Fangda Leng, Paul Edison

**Affiliations:** ^1^ Imperial College London, London United Kingdom

## Abstract

**Background:**

Pathological alterations in Alzheimer’s disease (AD) begin several years before the onset of symptoms. Grey matter mean diffusivity (MD) may be used as a measure of early grey matter damage in AD as it reflects the breakdown of microstructural barriers, which precede volumetric changes and affect cognitive function. In this study, we investigated the changes in MD early on in the disease trajectory, and relate them to the amyloid and tau deposition.

**Method:**

We analysed multimodal PET, DTI and MRI data of 90 participants, stratified into amyloid‐β negative and positive, cognitively normal (CN), mild cognitive impaired (MCI) and AD patients.

**Result:**

We found that MD was significantly increased in Aβ‐positive MCI and AD compared with CN in frontal, parietal, temporal cortex, hippocampus, and medial temporal lobe. We also found that cortical MD is significantly correlated with cortical thickness only in patients without amyloid deposition and is not significant in Aβ‐positive patients.

**Conclusion:**

Our results suggest that cortical MD is an early marker of neuronal damage as it is observed simultaneously as Aβ deposition and as it is correlated with cortical thickness when there is no Aβ deposition. This suggests that cortical MD provides information about early microstructural changes before macrostructural changes.

